# Spatio-Temporal Distribution of the Sources of Acoustic Events in Notched Fiber-Reinforced Concrete Beams under Three-Point Bending

**DOI:** 10.3390/ma16145118

**Published:** 2023-07-20

**Authors:** Dimos Triantis, Ilias Stavrakas, Andronikos Loukidis, Ermioni D. Pasiou, Stavros K. Kourkoulis

**Affiliations:** 1 Electronic Devices and Materials Laboratory, Department of Electrical & Electronics Engineering, University of West Attica, 250 Thivon Avenue, 122 44 Athens, Greece; ilias@uniwa.gr (I.S.); a.loukidis@uniwa.gr (A.L.); 2 Laboratory for Testing and Materials, Department of Mechanics, School of Applied Mathematical and Physical Sciences, National Technical University of Athens, Zografou Campus, 157 73 Athens, Greece; epasiou@central.ntua.gr (E.D.P.); stakkour@central.ntua.gr (S.K.K.)

**Keywords:** acoustic emissions, acoustic sources, plain concrete, fiber-reinforced concrete, three-point bending

## Abstract

The acoustic activity, generated in notched, beam-shaped concrete specimens, loaded under three-point bending, is studied in terms of the position of the sources of acoustic events, and the frequency of their generation. Both plain specimens (without any internal reinforcement) and specimens reinforced with various types of short fibers were tested. The target of the study is to investigate the existence of indices that could be considered as pre-failure indicators of the upcoming fracture. In addition, an attempt is undertaken to classify the damage mechanisms activated to tensile or shear nature. Considering comparatively the spatio-temporal evolution of the position of the acoustic sources and the respective temporal evolution of the frequency of generation of acoustic events, it was concluded that for relatively low load levels the acoustic sources are rather randomly distributed all over the volume of the specimens. As the load increases toward its maximum value, the acoustic sources tend to accumulate in the immediate vicinity of the crown of the notch and the average distance between them approaches a minimum value. When this minimum value is attained, the load is maximized and the generation frequency of the acoustic events increases rapidly. The simultaneous fulfillment of these three conditions is observed a few seconds before the onset of propagation of the catastrophic macrocrack for all classes of specimens tested, providing a kind of warning signal about the upcoming fracture. Moreover, the classification of the damage mechanisms to tensile and shear ones revealed a crucial difference between the plain and the reinforced specimens after the maximization of the load applied. Indeed, while for the plain specimens, the prevailing damage mechanism is tensile microcracking, for the reinforced specimens a balance between tensile and shear damage mechanisms is observed after the load applied has attained its peak and starts decreasing.

## 1. Introduction

As it is experimentally observed, elastic waves are generated at the interior of materials while they are subjected to increasing mechanical loading. These elastic waves are detected as acoustic signals by means of proper sensors and are known as Acoustic Emissions (AEs). The AEs are related to the onset of formation, growth, and coalescence of microcracks. It is beyond any doubt that proper analysis of AEs provides interesting information concerning the level of damage accumulation within the loaded material. In addition, it provides useful indices (known as pre-failure indicators), related to the entrance of the specimen (or the structure) into its critical loading stage, namely, the stage of impending catastrophic fracture.

Various characteristics and statistical parameters of the acoustic activity (for example, the frequency of generation of acoustic events, the energy release rate, the b-value, etc.), have been proven to be quite valuable and flexible tools, for the investigation of the evolution of microcracking processes within the loaded material (especially at the stage at which macroscopic fracture is approaching [1,2,3,4,5,6,7]), providing, in addition, reliable pre-failure indicators [8,9,10,11,12,13]. Moreover, it has been proven that the combined consideration of the Rise Angle (RA) and the Average Frequency (AF) of an acoustic signal permits the classification of the damage mechanisms activated to tensile or shear nature [14,15]. Intensive research activity is nowadays in progress in the direction of clarifying the interconnection between the AEs and the respective electric/electromagnetic activity (in the form of electric currents [16,17,18], or of electromagnetic emissions [19,20,21], or of variations of the electric resistance [22,23,24]), which is detected while specific classes of building materials are subjected to various mechanical loading schemes.

Along the same lines, the simultaneous use of more than one acoustic sensor (depending on the geometry of the loaded specimen) during the experimental procedure, permits the determination of the spatial coordinates (x, y, z) of the source of an acoustic event. In other words, it makes it possible to localize the points where the damage mechanisms are activated, at any time instant from the onset of loading up to the final macroscopic fracture. The spatial evolution of the position of the sources of the acoustic events has been used as a tool for the localization of the areas of intense microcracking activity by many researchers worldwide [1,25,26,27,28], as well as an index for the quantification of the degree of spatial concentration or dispersion (or, in other words, of the spatial “density”) of the sources of microcracking [29,30].

In general, acoustic events are detected during the whole loading procedure, already from relatively low load levels (compared to the loads causing macroscopic catastrophic fracture). In most cases, the acoustic events detected at very early loading steps are not directly related to the final fracture. In fact, there is a randomness in the spatial distribution of these early acoustic events, strongly related to the initial mechanical properties of the material, mainly its (an)isotropy and (in)homogeneity, pre-existing discontinuities, and the respective local stress fields around them. Clearly, the successful prediction of a macroscopic fracture depends on some knowledge of the density of these pre-existing “anomalies” of the structure of the material [31].

The spatial distribution of the acoustic events and other characteristics of the signals that are collected during the loading of a specimen until its fracture have been studied thoroughly and attempts are made to classify the results with respect to the remaining life of the specimen. The theory of “three stages” proposed by Mogi [26] is the one most widely adopted, and can be briefly described as follows: During the first stage, the acoustic activity is weak, and critical acoustic events are not detected or they are very rare. In the second one, the rate of generation of acoustic events appears relatively increased; however, their sources are still randomly distributed all over the mass of the loaded specimen. Finally, in the third stage, the sources of the acoustic events exhibit strongly increased concentration in the close vicinity of the points from which macroscopic cracks will start propagating. During this last stage, the frequency of generation of acoustic events is strongly intensified, and assuming that it is described in terms of the “time-to-failure” parameter, it is governed by a specific power law [32]. Moreover, the average Euclidean distance between the acoustic sources is proven to significantly decrease, while the level of the load applied tends to attain the critical level that causes macroscopic fracture [30].

In this direction, an attempt to quantify the spatio-temporal evolution of the position of the sources of acoustic events is undertaken in this study. To achieve this target, a novel approach is adopted, based on the average position of the sources of the acoustic events, which belong to a sliding window of n successive events. In parallel, for each “window” (group of acoustic events), the average frequency of generation of events is calculated in an attempt to enlighten relative correlations, which could be valuable for the detection of pre-failure indices. For the implementation of the above goals, advantage is taken of the data of an experimental protocol with beam-shaped notched specimens made of concrete, fiber reinforced or plain (i.e., without any kind of reinforcing elements), which were subjected to bending. The aforementioned analysis of the experimental data indicated that a series of conditions are simultaneously fulfilled a few seconds before the onset of propagation of the fatal macrocrack, suggesting that a proper combination of them could serve as an interesting pre-failure signal. The innovative aspect of the present study is the simultaneous and comparative study of the spatial and the temporal evolution of the position of the sources of acoustic events. To achieve this target, a novel experimentally determined parameter is introduced, representing the average Euclidean distance between the sources of the acoustic signals, i.e., the sources of damage mechanisms. The temporal evolution of this parameter is proven to provide valuable insight into the process of damage accumulation within the mass of the loaded specimen. Equally important innovative aspect of the study is the spatial localization of the average position of the acoustic sources with respect to the crown of the notch. It is the simultaneous analysis of these two parameters that permits detection of pre-failure indicators, namely, signals which are assumed to timely warn about the upcoming macroscopic fracture.

## 2. The Specimens and the Experimental Protocol

The protocol from which the experimental data were obtained has been analytically described in a previous paper by the authors’ team [12], and thus it is only synoptically outlined here. It included three-point bending experiments with specimens made of concrete. The specimens were beam-shaped of length equal to 700 mm and their cross-section was square (150 mm × 150 mm). All specimens were mechanically notched at their central section, using a rotating cutting disc. The depth of the notch was equal to 25 mm and its breadth (width) was equal to about 5 mm. The main role of the notch is to create an area of severe stress concentration, and thus to predefine the area where fracture will appear. Moreover, the existence of the notch enhances the activation of damage mechanisms from relatively early loading stages due to the locally intense stress field. Clearly, the novel parameters introduced in this study (i.e., the average Euclidean distance between the sources of the acoustic signals, and the average position of the acoustic sources with respect to the crown of the notch) could be equally well employed for the analysis of data obtained from protocols with intact beams.

Four different compositions of the concrete mixture were considered as shown in Table 1. The protocol was initially designed to include sixteen experiments (four specimens per composition); however, only fourteen were implemented successfully. 

The four specimens that will be described analytically in the next sections are typical ones for each composition. The response of the remaining specimens is more or less similar to that of the typical specimens chosen per composition.

It is noted at this point that the data of this protocol have been analyzed in previous works according to approaches based on quite different physical principles. It is mentioned, for example, that in ref. [8] the data were analyzed based on concepts of the “Natural Time Domain” (an approach which ignores the conventional time), while in ref. [12] the analysis is based on concepts of “Non-Extensive Statistical Mechanics” (founded on a class of entropies which violate the additivity property, namely, the property constituting the foundation of the familiar Boltzmann–Gibbs Statistical Mechanics—these entropies are commonly denoted as non-additive entropies).

**Figure 1:**
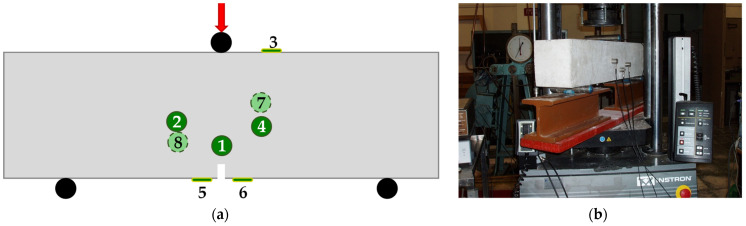
(**a**) The spatial arrangement of the eight acoustic sensors; dashed lines and light green color indicate sensors mounted at the rear face of the specimens; (**b**) a typical specimen before loading.

The specimens were supported with the aid of two metallic cylinders (Figure 1) and the load was applied at the central section (i.e., the notched one) by means of a third identical metallic cylinder. An Instron-Satec servo-hydraulic frame was used to apply the load, adopting displacement-control conditions at a rate of 0.08 mm/min. The acoustic activity was detected using eight acoustic sensors (R15a), the spatial arrangement of which is shown in Figure 1a. A characteristic photo of a specimen before loading is shown in Figure 1b.

It is mentioned at this point that, although eight acoustic sensors were used (rendering possible the determination of all three spatial coordinates of each acoustic source), the following analysis is implemented in a two-dimensional sense (in the present study in the (x, y) plane). In other words, the results are projected on the vertical plane of symmetry including the longitudinal axis of the beams. This is a first approach based on the assumption that the role of the third dimension (in the present study of the z-direction) is of minor importance, compared to that of the remaining two dimensions. Although this assumption sounds arbitrary, one should recall that, according to the elementary Bernoulli–Euler bending theory, the stress field developed is, in fact, two dimensional. Clearly, the validity of this assumption is to be further validated by means of additional protocols.

**Figure 2:**
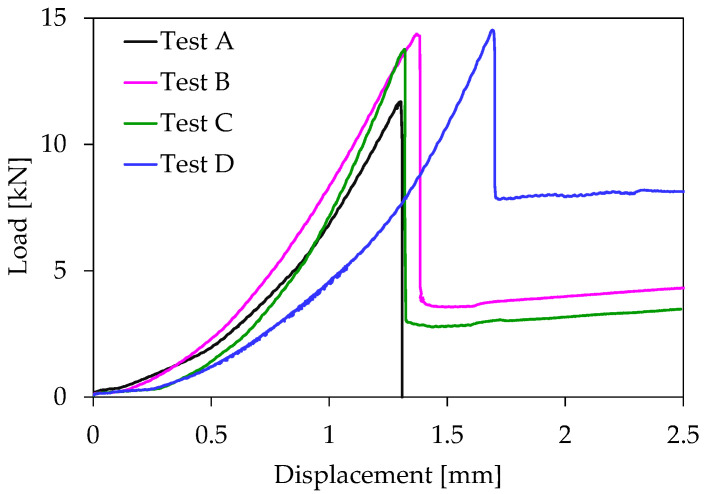
The mechanical response of typical specimens: The load applied versus the displacement.

The mechanical response of four typical specimens (one from each class of Table 1) is shown synoptically in Figure 2, in which the load applied is plotted versus the displacement induced. Excluding the specimen made of plain concrete (test A), which was fractured abruptly and the load was zeroed suddenly, the remaining three types of specimens failed according to a different manner: After attaining its peak value the load was reduced to a level equal to about 30% to 60% of the respective peak value, and then it was kept almost constant. In other words, the macroscopic crack that emanated from the crown of the notch did not propagate instantly toward the upper face of the beams, but rather it started propagating smoothly and slowly, due to the action of the fibers that kept together the two fragments, assigning a kind of significant “ductility” to the response of the beams.

It is to be mentioned here, that from the Strength of Materials point of view, there is no statistically significant difference between the values of the maximum load, L_max_, recorded for the three classes of fiber-reinforced specimens (specimens B, C, D). It is, perhaps, surprising that the role of the characteristics of the reinforcing fibers is rather marginal, at least concerning the strength of the product. However, this is by no means a definite conclusion. It is valid exclusively for the specimens of the present protocol and the authors did not attempt to explain it, since the mechanical response of the specimens was well beyond the scope of this study. In general, concerning the characteristics of the fibers, it is known that they play a crucial role, especially regarding the mechanical response and strength of the final product. However, the purpose of the present study was to analyze the acoustic activity in terms of alternative parameters (for example, the average distance between the acoustic sources and the average position of them), rather than to analyze the mechanical characteristics of the four classes of specimens. In this direction, it was not considered crucial to choose reinforcing fibers with specific characteristics.

## 3. The Methodology Adopted to Study the Spatio-Temporal Evolution of the Average Position of the Acoustic Sources

**Figure 3:**
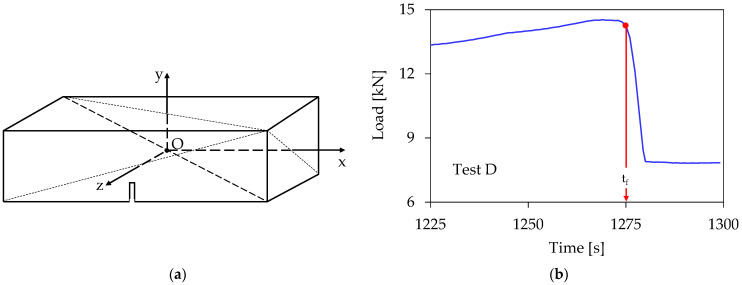
(**a**) The reference system adopted; (**b**) defining the conventional fracture instant for the reinforced specimens.

Taking advantage of the data simultaneously recorded by the eight acoustic sensors, and considering a reference system with its origin at the center of symmetry of the beam (see Figure 3a), the coordinates (x_i_, y_i_) of the sources of each acoustic event were determined, starting from the onset of the loading procedure up to the instant of macroscopic fracture, t_f_. It is clarified here that for the reinforced specimens (for which fracture is not instantaneous but rather the crack propagates slowly), a conventional fracture instant is defined, as the instant at which the load drops from its peak value to the value at which it is kept constant, as it is seen in Figure 3b, for a typical specimen of the fourth class.

The number of acoustic events, N, recorded for each one of the tests that will be analyzed in this study is shown in Table 2, along with the respective maximum load attained, L_max_. The N acoustic events for each experiment are divided into k groups, each one of which includes n events, where (for the needs of the present study) it was set n = 10 (the only exception is the last two or three groups, which may include n = 9 or n = 11 events, depending on the value of the N/k ratio, in the case that it is not an integer).

In order to study the spatio-temporal evolution of the average position of the acoustic sources in the vertical plane Oxy, the coordinates (x_i_, y_i_) of all acoustic sources, detected during the specific experiment, are retrieved from the software of the AE set up and the Euclidean distance between any two successive acoustic sources is determined as:

(1) $$D_{i-1}=\sqrt{{\left(x_{i}-x_{i-1}\right)}^{2}+{\left(y_{i}-y_{i-1}\right)}^{2}},\;\;\;\;\;\;i=2,\;\;3,\;\;\ldots$$

For each one of the k groups defined above, the average distance ${\bar{D}}_{j}$ between the acoustic sources (of the specific group) is determined as the mean value of the (n − 1) distances between the n successive sources of the group:

(2) $$\bar{D_{j}}=\frac{D_{1}+D_{2}+\ldots +D_{n-1}}{n-1},\;\;\;\;\;\;\;j=1,\;2,\;\;\ldots ,\;\;k$$

Finally, the average value of the coordinates of the acoustic sources for each one of the k groups is calculated as:

(3) $${\bar{x}}_{j}=\frac{x_{1}+x_{2}+\ldots +x_{n}}{n},\;\;\;\;{\bar{y}}_{j}=\frac{y_{1}+y_{2}+\ldots +y_{n}}{n},\;\;\;\;j=1,\;2,\;\;\ldots ,\;k$$

considering that each AE event (and thus each acoustic source) comprises many AE hits in the form of clusters. Therefore, using average values for x and y appears to be more appropriate in the direction of highlighting the evolution of the position of the AE sources inside the material and the way they “travel” around the notch’s crown. In addition, the average frequency ${\bar{f}}_{j}$ of generation of acoustic events is calculated for each one of the *k* groups, as:

(4) $${\bar{f}}_{j}=\frac{n}{\Delta t},\;\;\;\;\;\;j=1,\;\;2,\;\;\ldots ,\;\;k$$

where Δt is the time interval between the first and last acoustic event of the specific group.

As a final step, each one of the ${\bar{x}}_{j}$, ${\bar{y}}_{j}$, ${\bar{f}}_{j}$, and ${\bar{D}}_{j}$ (j = 1, 2, …, k) values is paired to an average time instant τ_j_, which is the average value of the time instants, t_i_, at which each one of the n events of the specific group was recorded.

(5) $$\tau_{j}=\frac{t_{1}+t_{2}+\ldots +t_{n}}{n},\;\;\;\;\;\;\;j=1,\;\;2,\;\;\ldots ,\;\;k$$

## 4. Results and Discussion

In this section, the acoustic activity generated in four typical specimens (one for each class) is studied according to a common pattern. As a first step, the temporal evolution of the frequency of generation of acoustic events is considered. Then, the temporal evolution of the average Euclidean distance between the acoustic source is analyzed. Both quantities are plotted versus the time-to-failure parameter, in juxtaposition to the respective evolution of the load applied. Finally, the spatial evolution of the acoustic sources (with respect to the crown of the notch) is plotted and discussed.

### 4.1. The Unreinforced Concrete (3pb-ref-b02)

**Figure 4:**
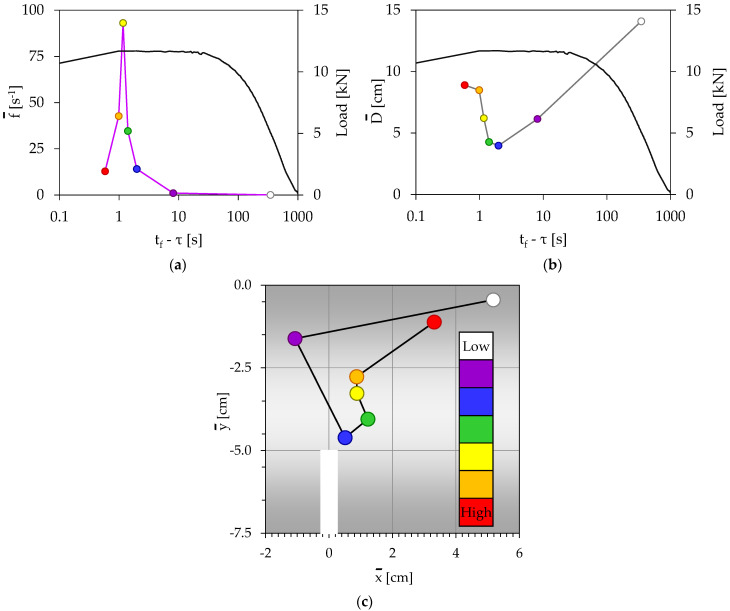
The temporal evolution of (**a**) the frequency of generation of acoustic events, and (**b**) the average Euclidean distance between the acoustic sources, in juxtaposition to the respective evolution of the load applied. (**c**) The spatial evolution of the acoustic sources (specimen without reinforcing fibers).

For the specific experiment, the N = 71 acoustic events recorded during the loading procedure, were divided into k = 7 groups (with 10 events for the first six of them and 11 events for the last one). The temporal evolution of the load applied is plotted in Figure 4a, in juxtaposition to the frequency of generation of acoustic events. Each group is colored with a different color, and the specific color code is kept constant for all figures describing the specific test.

Three time intervals can be distinguished in Figure 4a: During the first one (t_f_ − τ > 2 s), which covers the whole period of gradual increase in the load applied (until it attains its maximum value), the specimen appears to be completely “silent” concerning the acoustic activity, with $\bar{f}$ equal to almost zero (for t_f_ − τ > 9 s), or the acoustic activity is very weak (2 s < t_f_ − τ < 9 s) with $\bar{f}$ < 12 s^−1^. The first three groups of events (colored white, magenta, and blue) belong to this interval, in which the load is an increasing time function.

The second time interval (1 s < (t_f_ − τ) < 2 s) is characterized by a very steep increase in the frequency of generation of acoustic events, from $\bar{f}$ = 12 s^−1^ to $\bar{f}$ = 90 s^−1^, while the load applied is kept almost constant and equal to the peak value attained (L_max,A_ = 11.7 kN). Two groups of acoustic events (green and yellow) belong to this second interval.

Finally, during the third interval (0.4 s < (t_f_ − τ) < 1 s), both the frequency of generation of acoustic events and the load applied exhibit a decreasing trend until the specimens are macroscopically fractured. The last two groups of acoustic events (orange and red) belong to this third interval.

Concerning the temporal evolution of the average distance between the acoustic sources, which is plotted in Figure 4b, it is clearly seen that at the early loading stages its value is relatively high (it attains a maximum equal to about 14 cm), indicating that any damage mechanism activated (microcracking, debonding, fiber cracking etc.) produces results which are not as yet accumulated around specific material points of the specimen. Therefore, macroscopic failure is not impending. With the increasing load level, the average distance between the acoustic sources decreases toward a minimum value equal to about 4 cm, attained at about 2 s before fracture. Therefore, it is indicated that at this time instant the results of the damage mechanisms have been accumulated in the immediate vicinity of the crown of the notch (as it can be definitely concluded, also, from Figure 4c), rendering coalescence of microcracks significantly easier, and thus macroscopic cracking is now impending. After this critical time instant, the average distance between the acoustic sources starts increasing again, almost until the fracture of the specimen.

The spatial evolution of the average position of the acoustic sources is plotted in Figure 4c. It is clear from this figure that during the first time interval (t_f_ − τ > 2 s), the acoustic sources are randomly distributed and their average position is relatively far from the crown of the notch. As the frequency of generation of acoustic events starts increasing very rapidly (second time interval, 1 s < (t_f_ − τ) < 2 s), the acoustic sources appear as being attracted by the crown of the notch. Their average distance from the crown of the notch tends to be a minimum, attained at the third group of acoustic events. Then, the distance of the acoustic sources from the crown of the notch starts increasing again, maximized at the last group of events, i.e., just before the fragmentation of the specimen.

Comparative consideration of Figure 4a–c reveals that the time instant at which the average position of the acoustic sources (considered in the vertical axial plane of symmetry) is closer to the crown of the notch corresponds to:

(i) Maximization of the load applied;
(ii) Minimization of the distance between the acoustic sources;
(iii) Onset of rapid increase in the frequency of generation of acoustic events.

All these three conditions are simultaneously satisfied (for the specific tests) during the third group of events, which is “located” about 2 s before the macroscopic fracture. Alternatively, it can be said that the simultaneous fulfillment of these three conditions designates the onset of propagation of the fatal crack that will split the specimen into two fragments. During the last interval (in fact, during the last second before fragmentation), the acoustic activity seems to become significantly weaker in terms of the evolution of the average frequency of generation of acoustic events.

The above-described response can be attributed to several co-existing reasons. Indeed, at this last time interval, the failure plane is already “formed” (during the previous loading stages and due to the geometry of the specimens and the loading scheme adopted) and it is characterized by a local stress field with quite intense stress concentrations. Therefore, it is reasonable to assume that the AE activity at remote locations of the specimen (with respect to the fracture plane) is significantly reduced since the energy is mainly consumed around the tip of the macrocrack that has already started propagating. Moreover, the damages within and close to the fracture plane are very severe, and thus the elastic energy released due to any event is very high, reducing the energy locally available for an additional event to be generated (increasing simultaneously the time interval required for this generation). This behavior could explain the experimentally observed high energy and long duration of the acoustic events recorded during the last loading stages. Similar behavior to that described here has been observed, as well as in recent works of the team of authors [12,23], in which completely different methods of analysis of the acoustic activity were adopted.

### 4.2. Concrete Reinforced with Short Polyolefin Fibers (Force-60-4-b02)

**Figure 5:**
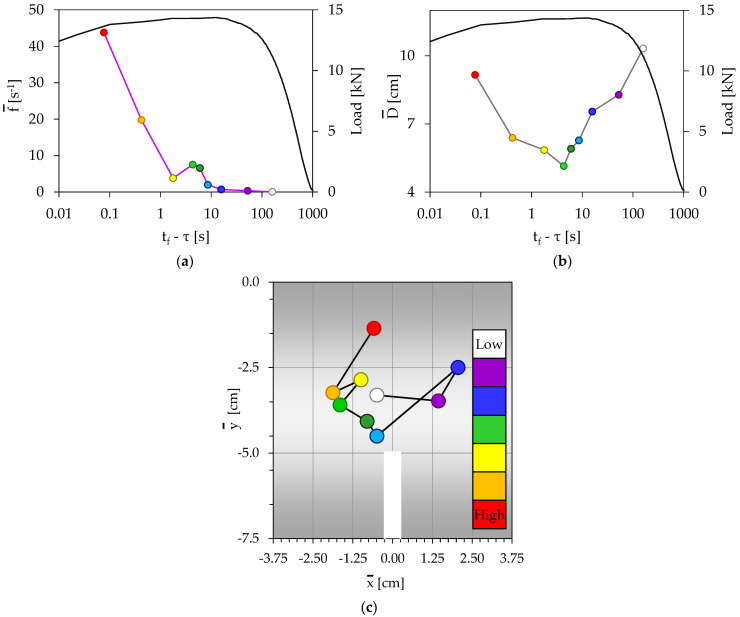
(**a**) The temporal evolution of (**a**) the frequency of generation of acoustic events, and (**b**) the average Euclidean distance between the acoustic sources, in juxtaposition to the respective evolution of the load applied. (**c**) The spatial evolution of the acoustic sources (specimen with polyolefin reinforcing fibers).

During the loading procedure of this experiment, N = 89 acoustic events were recorded. They were divided into k = 9 groups with n = 10 events for each one excluding the 9th group which contained only nine events. The temporal evolution of the load applied is plotted in Figure 5a versus the time-to-failure parameter (t_f_ − τ), in juxtaposition to the frequency of generation of acoustic events. As previously mentioned, each group is marked with a different color, which is kept constant for all figures devoted to this test.

Consistently with the previous test, the acoustic activity is negligible, almost for the whole period during which the load applied is increasing, i.e., for the interval t_f_ − τ > 8 s. The first three groups (white, magenta, and blue) of events belong to this interval. Then, while the load has already attained its maximum value (L_max,B_ = 14.37 kN), the acoustic activity starts increasing and the frequency of generation of acoustic events attains values approaching 8 s^−1^. During this interval (which includes three groups of acoustic events, namely, the light blue, the green, and the light green ones), the duration of which is equal to 4 s (4 s < t_f_ − τ < 8 s), the load applied has exceeded its peak value and it has started decreasing, although imperceptibly, from 100% to 99.6% of L_max,B_.

For both these intervals, i.e., for t_f_ − τ > 4 s, the temporal evolution of the average distance between the acoustic sources, which is plotted in Figure 5b, exhibits a steadily decreasing trend, i.e., the acoustic sources are approaching each other: the average distance between them decreases from a relatively high value, equal to about 10.5 cm, to a global minimum value equal to about 5 cm.

During the last 4 s of the loading procedure, i.e., for t_f_ − τ < 4 s, the distance between the acoustic sources starts increasing again, while the acoustic activity keeps intensifying after a relatively small drop. The specific behavior is explained by taking into account that although the macrocrack has started propagating at about t_f_ − τ ≈ 4 s, the presence of reinforcing fibers does not permit abrupt fragmentation of the specimens. Therefore, the crack propagates very slowly and additional events are now generated due to two additional reasons: Fracture of fibers which keep the two parts of the specimen together and shear effects along the fibers–mortar interfaces.

The spatial evolution of the average position of the acoustic sources is plotted in Figure 5c. It is seen that for the three first groups of acoustic events (corresponding to negligible or weak acoustic activity, i.e., for t_f_ − τ > 8 s), their sources are rather randomly distributed. At the instant that the acoustic activity is intensified (fourth group of acoustic events, or t_f_ − τ ≈ 4 s) the average distance of acoustic sources from the crown of the notch is minimized, indicating clearly that the macrocrack starts propagating. During the last interval of the loading procedure (t_f_ − τ < 4 s), the distance of the acoustic sources from the crown of the notch starts increasing again; however, it remains relatively close to the plane of symmetry, since the acoustic events are now generated due to failure (fracture or relative slip) of fibers within the plane of symmetry (i.e., of fibers that keep the two fragments together while the crack propagates slowly toward the upper surface of the specimen).

Considering, again, comparatively Figure 5a–c, similar conclusions are drawn with those obtained from the analysis of the unreinforced specimen: The time instant at which the average position of the acoustic sources (considered in the vertical axial plane of symmetry) is closer to the crown of the notch corresponds to maximization of the load applied, a tendency of minimization of the distance between the acoustic sources and the onset of increase in the frequency of generation of acoustic events. In this test, all these three conditions are satisfied at an instant with t_f_ − τ ≈ 4 s. The only difference between this test and that with the unreinforced specimen is that the acoustic activity keeps increasing even during the very last loading stage, i.e., after the macrocrack has started propagating; however, the mechanisms responsible for this activity are related to the failure of the fibers in the transverse vertical plane of symmetry, namely, the fibers that prevent abrupt fracture of the beam.

### 4.3. Concrete Reinforced with Short Polypropylene Fibers (pp940 50 6 b02)

**Figure 6:**
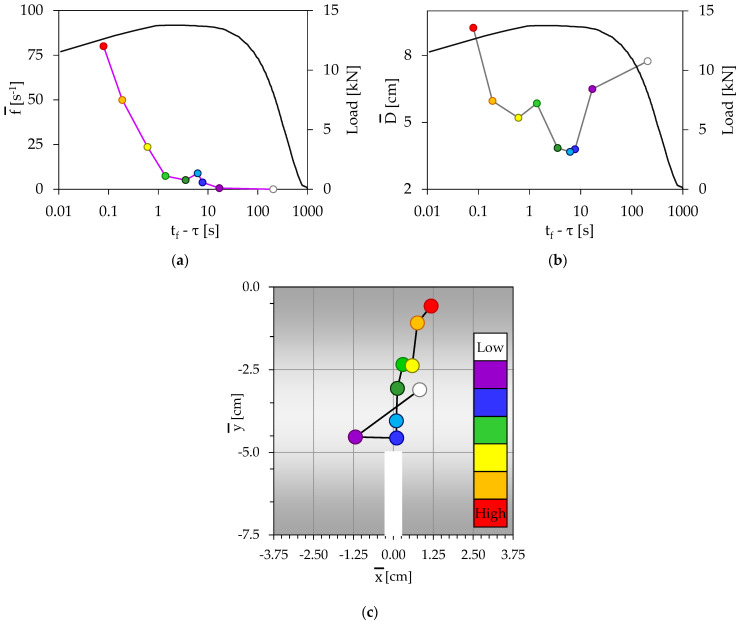
(**a**) The temporal evolution of (**a**) the frequency of generation of acoustic events, and (**b**) the average Euclidean distance between the acoustic sources, in juxtaposition to the respective evolution of the load applied. (**c**) The spatial evolution of the acoustic sources (specimen with polypropylene reinforcing fibers).

In this experiment, typical for the class of specimens reinforced with short polypropylene fibers, N = 87 acoustic events were recorded. They were divided, again, into k = 9 groups with n = 10 events for the first six of them and n = 9 events for the last three groups of acoustic events. The same as in previous quantities (i.e., the temporal evolution of the load applied, that of the frequency of generation of acoustic events, and that of the average Euclidean distance between the acoustic sources as well as the spatial evolution of the acoustic sources) are shown in Figure 6. It is clear that the response of this specimen is absolutely compatible, if not identical (with minor differences of quantitative nature), with that of the previous section (namely, the specimen with reinforcing fibers made of polyolefin): The acoustic activity is negligible or very weak almost until the load attains a value closely approaching the maximum value attained (L_max,C_ = 13.75 kN), i.e., a value equal to about 99.5% of L_max,C_ (t_f_ − τ > 8 s). The average distance between the acoustic sources is steadily decreasing toward a global minimum value, which is finally attained at t_f_ − τ ≈ 6 s, the instant at which the acoustic activity attains its local maximum (light blue group). Then, exactly as in the previous specimen, both the acoustic activity and the distance between the acoustic sources keep increasing, for the same previous reasons (failure due to fracture or slip of the fibers in the transverse vertical plane of symmetry, i.e., of the fibers that prevent abrupt fracture, despite the fact that the macrocrack is already propagating although very slowly).

Moreover, the spatial distribution of the acoustic sources follows the same pattern: The sources are initially randomly distributed and gradually they are attracted by the crown of the notch, attaining their smallest distance at the onset of the intensification of the acoustic activity (blue group of events), i.e., at about t_f_ − τ ≈ 6 s. As mentioned previously, from this instant on, the acoustic sources are moving away from the crown of the notch, remaining within the transverse plane of symmetry (i.e., the plane of fracture), following, in fact, the front of the slowly propagating (due to the action of the reinforcing fibers) macrocrack.

All three conditions (mentioned for the previous two specimens), which are fulfilled while the distance of the acoustic sources from the crown of the notch tends to be minimized (thus triggering the onset of propagation of the fatal macrocrack), i.e., maximization of the load, minimization of the distance between the acoustic sources and onset of rapid increase in the frequency of generation of acoustic events, are simultaneously satisfied at the instant t_f_ − τ ≈ 6 s.

### 4.4. Concrete Reinforced with Short Steel Fibers (Metal-25-B01)

During the last experiment of the protocol discussed in this study, N = 88 acoustic events were recorded. As mentioned previously, they were divided into k = 9 groups with n = 10 events for the first seven of them and n = 9 events for the last two groups.

**Figure 7:**
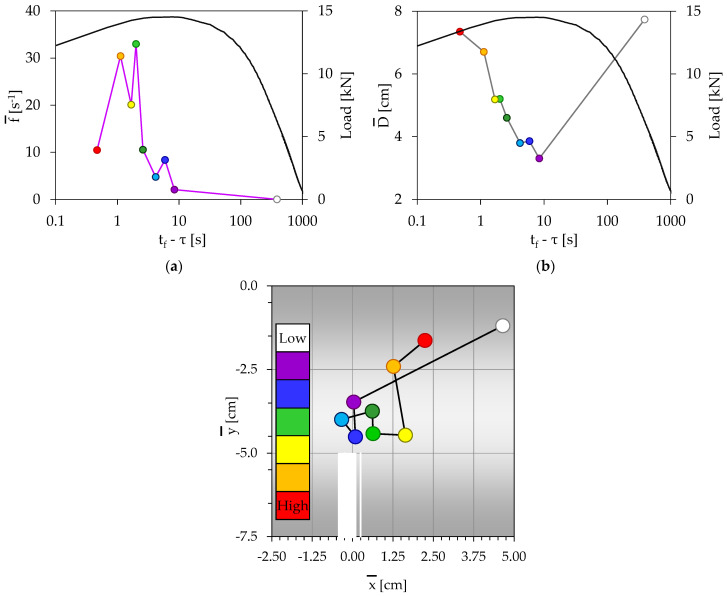
(**a**) The temporal evolution of (**a**) the frequency of generation of acoustic events, and (**b**) the average Euclidean distance between the acoustic sources, in juxtaposition to the respective evolution of the load applied. (**c**) The spatial evolution of the acoustic sources (specimen with steel reinforcing fibers).

The temporal evolution of the load applied is plotted in Figure 7a, in juxtaposition to the respective evolution of the frequency of generation of acoustic events. The evolution of the average Euclidean distance between the acoustic sources is shown in Figure 7b. Finally, the spatial evolution of the acoustic sources is depicted in Figure 7c. Although the response of the specific specimen exhibits considerable similarities with the previously discussed specimens reinforced with plastic fibers (made of polyolefin or polypropylene ones), these are some of the differences that should be highlighted:

- The first is that one detects now acoustic activity from relatively earlier loading steps, although it is again very weak for the t_f_ − τ > 8 s interval, during which the load applied attains 99.5% of its maximum value (L_max,D_ = 14.53 kN). The average distance between the acoustic sources decreases from a value equal to about 7.7 cm to a global minimum value equal to about 3.3 cm.
- The second difference in the response of the specific specimen (compared to the ones reinforced with plastic fibers) is that the acoustic activity does not increase monotonously up to the final fracture of the specimen. At about t_f_ − τ ≈ 2 s, a global maximum is attained and then the activity starts decreasing, although not monotonically, until the fracture instant. During both these periods (i.e., the period of rapid increase in the acoustic activity and the period of its rapid decrease), namely, for the interval t_f_ − τ < 8 s, the average distance between the acoustic sources exhibits an increasing trend from the global minimum value of 3.3 cm toward a maximum value approaching (for the last group of events) the value of 7.7 cm, which was attained during the very early loading steps (first group of events).

Concerning the spatial distribution of the acoustic sources, it follows exactly the same as the previous pattern: For the first group of events (t_f_ − τ < 8 s), the sources are relatively far away from the crown of the notch. Then, during the next two groups of events (magenta and blue), the acoustic sources are densely packed very close to the crown of the notch. This trend is terminated at the instant t_f_ − τ ≈ 6 s, corresponding to a local maximum of the acoustic activity. From this instant on, the acoustic sources are moving away from the crown of the notch; however, they remain within (or very close) to the transverse plane of symmetry (in other words, within the fracture plane), following again the front of the slowly (due to the presence of the reinforcing fibers) propagating macrocrack.

The three conditions, that were fulfilled for all previous specimens (reinforced or not) in order for the distance of the acoustic sources from the crown of the notch to be minimized (namely, the maximization of the externally applied load, the minimization of the distance between the acoustic sources, and the onset of the increase in the frequency of generation of acoustic events), are here simultaneously satisfied in a narrow time interval, with 6 s< t_f_ − τ < 8 s. Assuming that the instant, at which the sources of the acoustic events approach very closely the crown of the notch, is the triggering of the onset of propagation of the final catastrophic macrocrack, it can be safely stated that in this experiment the macroscopic fracture started at an instant between 6 and 8 s before the conventional fracture instant (as it was previously defined in Section 3).

## 5. Crack Classification Using the Relationship between the Average Frequency and RA Value

In this section, an attempt is undertaken to classify the microcracking activity as tensile or shear, in order to validate the previous assumption about the nature of the damage mechanisms activated before and after the applied load attains its maximum value. This classification is based on the individual hit waves, as recorded by the AE sensors, and projects a relation between the Average Frequency, AF, and the RA parameter (namely, the Rise Time (RT) per Amplitude) according to RILEM [33]. The Average Frequency (AF) is defined as the number of counts divided by the total duration of an acoustic hit and it is measured in kHz, while the RA value is defined as the Rise time divided by the peak Amplitude of an AE hit and is measured in ms/V.

According to this approach, mode-II damage mechanisms (frictional or equivalently shear mechanisms) correspond to acoustic signals with high RA and intermediate AF values. On the contrary, mode-I mechanisms (tensile microcracking) correspond to acoustic signals with low RA values combined with high AF ones. It is to be emphasized at this point, that in the original contributions, in which the AF to RA relation was introduced as a characteristic that distinguishes mode-I from mode-II acoustic events [14,15], a unique numerical value for the slope of the “dividing line” was not suggested. It is merely a choice of each researcher and, usually, it is assumed to be the diagonal line of the AF-RA plots, assuming that a ratio of the respective scales is equal to 10.

**Figure 8:**
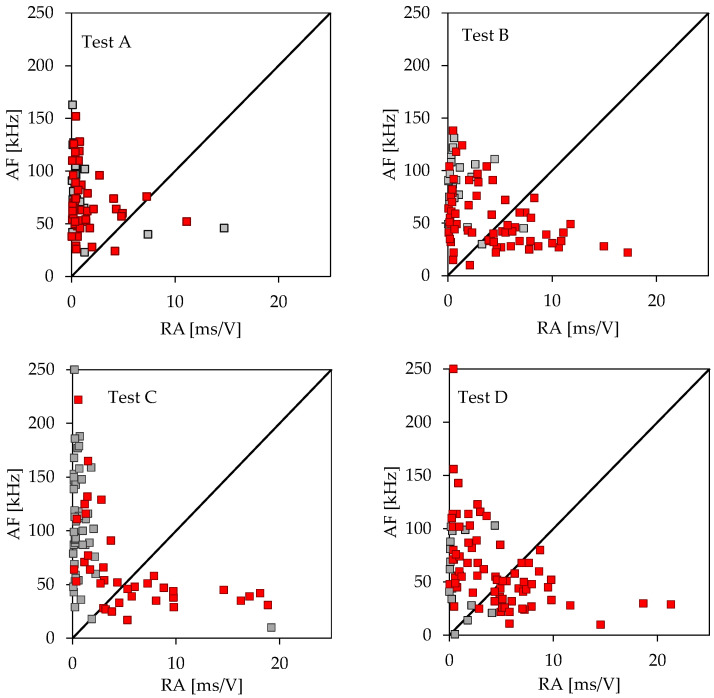
Classification of the acoustic signals detected as tensile (above the diagonal, the slope of which is equal to 10) and as shear (below the diagonal) for the four tests analyzed in Section 4 (grey rectangles correspond to Group I while red ones to Group II).

In the present study, two groups of acoustic hit signals are considered: The first one (denoted as Group I and represented here with grey rectangles) includes signals which are generated until the time instant at which the load attains its peak value, L_max_, while the second group (denoted as Group II, and represented here with red rectangles) includes the signals generated while the load imposed starts decreasing until the fracture (actual or conventional) of the specimen. The results of this classification for the four experiments described in the previous section are shown in Figure 8, while in Table 3 one can see the percentage of acoustic signals that belongs to each one of the two categories (i.e., shear or tensile) for both A and B groups.

It is clearly concluded from Figure 8 and Table 3, that for the unreinforced specimen, the vast majority of acoustic signals are classified as tensile, both before the load attains its peak value (about 92% of the total signals), and while the load starts decreasing (about 96% of the total signals). On the contrary, for all three types of reinforced specimens, this is true only before the maximization of the load applied: Indeed, during this time interval, the signals detected are classified as tensile (with a percentage ranging from 89% to 94%). However, after the load has been maximized and its value has started decreasing, the situation changes dramatically: About half of the signals generated in this interval are classified as shear ones, thus verifying the conclusions previously drawn in Section 4, that while the load decreases an additional damage mechanism (in addition to that of tensile microcracking) is now activated, namely, that of debonding of the reinforcing fibers from the concrete mass. Moreover, an almost perfect balance between the two damage mechanisms (i.e., tensile microcracking and debonding) is observed.

In order to gain a deeper insight into the gradual transition from the purely tensile damage mechanism to the combined action of the microcracking and debonding, an index m is introduced, defined as the ratio of the Average Frequency (AF) over the RA value of the “primary acoustic hit” of each event. In order to clarify the term “primary acoustic hit”, one should recall that any acoustic event is defined with the aid of a number of acoustic hits, recorded by a series of acoustic sensors. The first one of these hits, i.e., the one that arrived first at one of the sensors is characterized as the “primary acoustic hit”.

**Figure 9:**
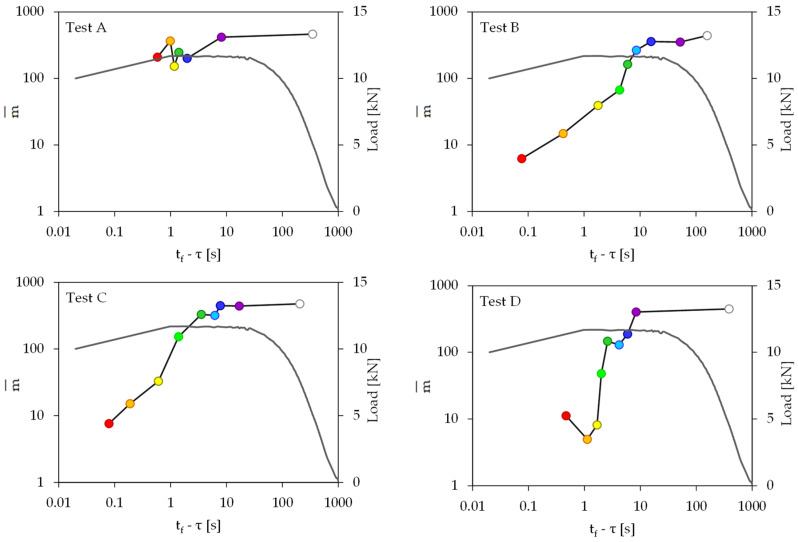
The temporal evolution of the index $\bar{m}$ in juxtaposition to the respective evolution of the load applied versus the (t_f_ − τ) parameter for all four experiments considered.

Considering the k groups of acoustic events (as it is analytically described in Section 4), one can determine the average value $\bar{m}$ of the index m for each one of the k groups of each experiment. The temporal evolution of the average value $\bar{m}$ of the index m is plotted in Figure 9 versus the (t_f_ − τ) parameter.

It is once again definitely concluded from Figure 9 that for the unreinforced specimen, the dominant damage mechanism activated throughout the whole loading procedure (i.e., from the onset of loading until the catastrophic fracture of the specimen) is the mode-I mechanism (namely, tensile microcracking). Indeed, for all k = 7 groups of acoustic events of this experiment, the average value of m attains extremely high values ranging in the 150 < $\bar{m}$ < 450 interval. The maximum value $\bar{m}$ ≈ 450 corresponds to the first group of events, while the minimum value $\bar{m}$ ≈ 150 corresponds to the fifth group of events (recall that this fifth group of acoustic events corresponds to the maximization of the intensity of the acoustic activity). 

On the other hand, for all three classes of reinforced specimens, the average value of the index m exhibits a constantly decreasing trend after the load has been maximized. Indeed, starting from an extremely high value (in the range of 400 < $\bar{m}$ < 500 for all three experiments) it approaches values even below $\bar{m}$ = 10, for the very last groups of acoustic events. In other words, after the load has attained its peak value, the mode-I damage mechanism recedes gradually in favor of the mode-II one. The consistency of the temporal evolution of the m-index for all three classes of reinforced specimens (independently of the nature of the reinforcing fibers) is striking.

## 6. Concluding Remarks

Notched, beam-shaped specimens, made of plain or fiber-reinforced concrete were loaded under three-point bending. The evolution of the acoustic activity generated during the loading procedure was considered both in terms of time and space. More specifically, the position of the acoustic sources and the average distance between them were comparatively studied in juxtaposition to the temporal evolution of the average frequency of generation of acoustic events.

It was concluded that independently of the nature of the specimens tested (plain or fiber reinforced), some features of the acoustic activity are common. Indeed, at the time instant at which the load applied tends to be maximized three conditions are, almost, simultaneously and systematically fulfilled:

i. The average distance between the acoustic sources attains a global minimum value and then it starts increasing again;
ii. The acoustic sources are densely packed in the immediate vicinity of the crown of the notch and then they start gradually moving away from the specific area;
iii. The frequency of generation of acoustic events starts increasing quite abruptly.

As it is expected, the acoustic activity is very weak at the very early loading stages (see, for example, Figure 4a, Figure 5a, Figure 6a and Figure 7a). Then, it is gradually intensified and the onset of its severe intensification is detected at the instant at which the average position of the acoustic sources from the crown of the notch is minimized (while simultaneously the distance between the acoustic sources is, also, minimized). After this instant, at least one macrocrack has started propagating producing “strong” acoustic signals, and thus “high” acoustic activity. In this context, Figure 4c, Figure 5c, Figure 6c, and Figure 7c can be considered as representing the location of the tip of the propagating macrocrack, explaining why the characterization “high” for the acoustic activity is associated with locations which are moving gradually away from the crown of the notch.

**Figure 10:**
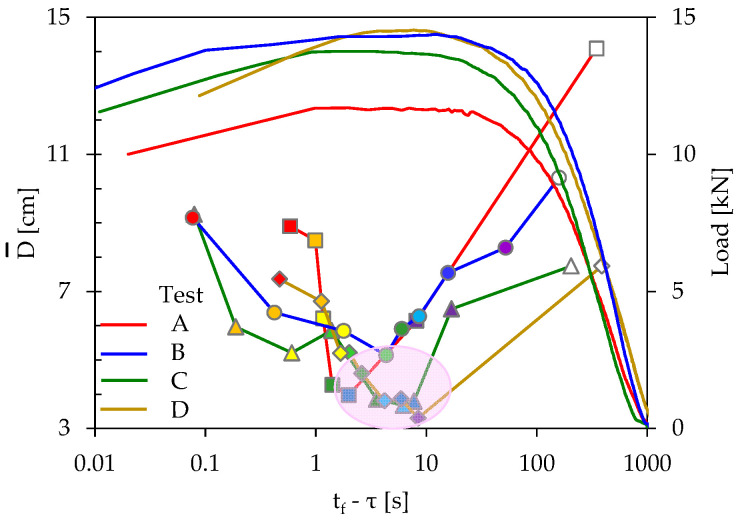
The temporal evolution of the average distance between the acoustic sources in terms of the time to-failure parameter, for all specimens analyzed, in juxtaposition to the respective evolution of the load applied.

The above conclusions are systematically observed for all specimens tested, and the differences between them are quantitative rather than qualitative. This is vividly seen in Figure 10, in which the average distance between the acoustic sources is plotted comparatively for all specimens analyzed in previous sections. It is clear that, despite the different compositions of the specimens, the Euclidean distance between the acoustic sources attains a global minimum, in a narrow time interval (ranging from about 2 s to 9 s before fracture). Moreover, the magnitude of this global minimum ranges within very narrow limits, i.e., in the range from 3.9 cm to 5.1 cm.

Of equal importance is the observation concerning the nature of the damage mechanisms activated, especially while the load applied approaches its maximum value.

- For the plain specimens, the damage mechanism prevailing is that of mode-I, i.e., tensile macrocracking, both before the load attains its peak value, and while the load enters its decreasing stage.
- For the reinforced specimens (with plastic or metallic fibers), the mode-I damage mechanism prevails only while the load is increasing. During the interval at which the load attains its maximum value and is kept almost constant (and during the time interval that it starts decreasing), acoustic events due to the mode-II damage mechanism are generated and gradually the two mechanisms reach an almost perfect balance between them. Clearly, the mode-II damage mechanism is attributed to the gradual debonding of the reinforcing fibers (that keep together the two parts of the beam on either side of the slowly propagating macrocrack) from the concrete matrix, while the mode-I events are attributed to the fracture of these fibers and not to microcracking within the body of the concrete matrix.

In conclusion, it was clearly highlighted that a series of conditions were systematically fulfilled simultaneously a few seconds (ranging from about 2 s to about 10 s) before the propagation of the fatal macrocrack. At the same time instant, the average distance of the position of the sources of acoustic hits from the crown of the notch attains its minimum value (ranging from 2.5 mm to 4.0 mm). These observations were systematically noticed for all the specimens tested (plain or reinforced), suggesting that each one of the conditions fulfilled could be considered as a reliable pre-failure index, or, at least, that this idea deserves to be further (and more thoroughly) studied for wider classes of materials and different loading protocols, including in situ Structural Health Monitoring, of large-scale structures. It should be, however, kept in mind that moving from “sterilized” laboratory conditions to in situ Structural Health Monitoring is a challenging step. Various types of “noises” may “infect” the acoustic signals recorded. The specific issue is among the most critical ones of in situ Structural Health Monitoring, and although huge effort is made worldwide to objectively “clean” the signals recorded, the issue is not as yet closed.

## Figures and Tables

**Table 1:** The four types of specimens tested.

Test	Sample/Code	Fiber’s Density	Characteristics of the Fibers
A	Plain concrete/ ref-b02	- - -	- - -
B	Concrete reinforced with short plastic fibers/ force60-4-b02	4 kg/m^3^	Polyolefin Length ≈ 60 mm, Diameter ≈ 0.84 mm Tensile strength ≈ 465 MPa
C	Concrete reinforced with short plastic fibers/ pp940-50-6-b02	4 kg/m^3^	Polypropylene Length ≈ 50 mm, Diameter ≈ 0.75 mm Tensile strength ≈ 600–650 MPa
D	Concrete reinforced with short metallic fibers/ metal-25-B01	25 kg/m^3^	Steel Length ≈ 50 mm, Diameter ≈ 1.30 mm Tensile strength ≈ 690 MPa

**Table 2:** The number of acoustic events recorded and the maximum load attained for each specimen.

Specimen	Test	N	L_max_ [kN]
3pb-ref-b02	A	71	11.70
force60-4- b02	B	89	14.37
pp940-50-6-b02	C	87	13.75
metal-25-B01	D	88	14.53

**Table 3:** The number of acoustic events recorded prior and after the maximization of the load applied for each test.

Test ↓	Group→	*Group I (up to L_max_)*		*Group II (after L_max_)*	
Type of Signal→		*Tensile*	*Shear*	*Tensile*	*Shear*
A		92%	8%	96%	4%
B		92%	8%	52%	48%
C		94%	6%	49%	51%
D		89%	11%	51%	49%

## Data Availability

Not applicable.
